# Supplementary material for: A Multifaceted Exploration of Status Asthmaticus: A Retrospective Analysis in a Romanian Hospital
Source: J Clin Med. 2024 Nov 4;13(21):6615. doi: 10.3390/jcm13216615 (PMC11546779; doi:10.3390/jcm13216615)
Supplement: Supplementary file 1 [file jcm-13-06615-s001.zip › jcm-3223062-supplementary.pdf]

Journal of *Personalized Medicine*

MDPI

Article

A Multifaceted Exploration of Status Asthmaticus: A Retrospective Analysis in a Romanian Hospital

Adriana Ana Trusculescu [1,2], Versavia Maria Ancusa [3,*], Camelia Corina Pescaru [1,2], Norbert Wellmann [1,2,4], Corneluta Fira-Mladinescu [5,6], Cristian Iulian Oancea [1,2] and Ovidiu Fira-Mladinescu [1,2]

[1] Center for Research and Innovation in Personalized Medicine of Respiratory Diseases (CRIPMRD), Pulmology University Clinic, 'Victor Babes' University of Medicine and Pharmacy, Eftimie Murgu Square no. 2, 300041 Timisoara, Romania; ana.trusculescu@umft.ro (A.A.T.); pescaru.camelia@umft.ro (C.C.P.); norbert.wellmann@umft.ro (N.W.); oancea@umft.ro (C.I.O.); mladinescu@umft.ro (O.F.-M.)
[2] Pulmology University Clinic, Clinical Hospital of Infectious Diseases and Pneumophysiology Dr. Victor Babeș Timișoara, Gheorghe Adam Street, no. 13, 300310 Timisoara, Romania
[3] Department of Computer and Information Technology, Automation and Computers Faculty, "Politehnica" University of Timis, Vasile Pârvan Blvd, no. 2, 300223 Timisoara, Romania
[4] Doctoral School, "Victor Babes" University of Medicine and Pharmacy Timisoara, Eftimie Murgu Square 2, 300041 Timisoara, Romania
[5] Hygiene Division, Department of Microbiology, "Victor Babes" University of Medicine and Pharmacy Timisoara, Victor Babes Street, no. 16, 300226 Timisoara, Romania; fira-mladinescu.corneluta@umft.ro
[6] Center for Study in Preventive Medicine, "Victor Babes" University of Medicine and Pharmacy Timisoara, Eftimie Murgu Square no. 2, 300041 Timisoara, Romania
* Correspondence: versavia.ancusa@upt.ro

Table S1. Comparison between regular asthma (J45) and status asthmaticus (J46) complex network parameters

	J45	J46
**#of Nodes:**	2231	174
**#of Edges:**	70708	2449
**Average Degree**	63.387	18.333
**Avg. Weighted Degree**	283.418	28.149
**Network Diameter**	3	2
**Radius:**	2	1
**Average Path length:**	2.041	1.8940
**Graph Density**	0.028	0.106
**Average Clustering Coefficient:**	0.828	0.836
**Total triangles:**	1813066	7063
**Modularity**	0.089	0.219
**No of classes:**	7	6
**Proportions:**	43.39%, 33.84%, 22.01%, 0.27%, 0.22%, 0.18%, 0.09%	34.48%, 20.69%, 18.97%, 14.37%, 9.2%, 2.3%

Table S2. J46 node list, sorted largest to smallest based on degree value

Label	Degree	Weighted Degree	Betweeness centrality	Modularity class
J46	173	493	0.386581	1
I10	114	275	0.10041	1
J96.0	108	292	0.094381	1
J45.8	93	229	0.059135	1
J96.1	87	165	0.059791	2
J18.9	75	176	0.032544	1

N39.0	66	136	0.028407	1
I11.0	52	97	0.009945	1
E66.0	51	96	0.009615	1
I50.0	48	76	0.011674	1
I25.5	47	83	0.007944	4
I34.0	47	76	0.010787	1
B37.0	45	73	0.011782	1
J15.8	39	52	0.009534	4
J31.0	36	70	0.004155	1
R74.0	36	45	0.004603	0
B96.88	35	56	0.00331	4
K76.0	35	45	0.003178	1
Z72.0	33	47	0.004906	3
Z99.1	33	44	0.002708	0
N18.90	31	36	0.002969	0
J44.0	30	47	0.004241	2
G20	30	32	0.001775	0
J84.9	29	51	0.004081	2
J45.9	29	45	0.002458	1
I48	29	31	0.001486	0
M47.84	28	43	0.001164	0
I50.9	28	36	0.00182	0
E11.9	28	33	0.000967	0
E78.0	26	42	0.003123	2
R73	24	35	0.002114	1
E87.1	24	26	0.000855	0
J44.1	23	37	0.002187	2
I25.9	23	35	0.001856	1
E79.0	23	27	0.000372	0
E87.6	23	26	0.000634	0
I83.1	23	25	0.000993	4
B90.9	22	29	0.002004	2
J43.9	22	29	0.002499	2
M54.4	21	30	0.001255	2
J84.1	21	23	0.001067	3
B37.1	20	36	0.000641	1
K81.1	20	24	0.000587	4
D75.2	20	20	0	0
I25.0	20	20	0	0
I44.7	20	20	0	0
I51.7	20	20	0	0
I11.9	19	26	0.000227	4
J45.0	19	22	0.00072	1
Z11.5	18	28	0.000957	0
E11.65	18	23	0.000733	4
J44.9	18	19	0.000862	3

D53.9	17	24	0.000845	2
I20.0	16	19	0.000413	3
I27.2	16	19	0.000253	3
D50.8	16	18	0.000415	4
K29.5	16	18	0.000223	3
E66.8	15	19	0.000148	1
J34.2	15	19	0.000149	1
H91.9	14	17	0.000325	2
E11.8	13	17	0.000133	4
C34.9	13	13	0	3
C78.1	13	13	0	3
E04.9	13	13	0	1
E07.8	13	13	0	3
I36.0	13	13	0	1
I70.0	13	13	0	4
J32.1	13	13	0	1
J82	13	13	0	1
K29.9	13	13	0	4
K44.9	13	13	0	3
K51.9	13	13	0	1
K92.8	13	13	0	4
M05.99	13	13	0	4
M15.9	13	13	0	4
M17.9	13	13	0	4
M30.1	13	13	0	1
M79.28	13	13	0	1
R59.0	13	13	0	3
Y43.4	13	13	0	4
A41.8	12	16	0.000215	2
J47	12	15	0.000197	2
E03.9	12	14	0.000198	1
E66.9	12	14	0.000249	2
B95.7	12	12	0	4
E06.3	12	12	0	4
F33.8	12	12	0	4
I07.1	12	12	0	4
I25.6	12	12	0	4
I87.8	12	12	0	4
M17.0	12	12	0	4
E09.01	11	18	0.000023	2
K25.9	11	18	0.000023	2
N40	11	18	0.000023	2
M47.82	11	14	0.00015	1
T78.4	11	13	0.000202	1
B96.2	11	11	0	4
I08.0	11	11	0	4

Z71.3	10	17	0.000016	1
I87.2	10	14	0.000012	1
J18.0	10	12	0.00009	1
B37.81	10	10	0	2
H05.2	10	10	0	0
I20.8	10	10	0	1
J12.9	10	10	0	3
J91*	10	10	0	4
K91.1	10	10	0	4
N11.0	10	10	0	3
Q33.1	10	10	0	0
R31	10	10	0	3
B96.5	9	9	0	2
B97.1	9	9	0	1
D69.0	9	9	0	1
D72.8	9	9	0	0
E78.2	9	9	0	0
H10.9	9	9	0	3
H35.9	9	9	0	3
H81.4	9	9	0	1
J94.1	9	9	0	2
J95.1	9	9	0	2
K76.9	9	9	0	0
Q61.3	9	9	0	0
T78.2	9	9	0	1
Z88.1	9	9	0	1
Z88.6	9	9	0	1
L30.9	8	14	0	1
R64	8	14	0	1
B95.6	8	8	0	2
D64.9	8	8	0	1
D69.6	8	8	0	2
H54.0	8	8	0	2
I49.3	8	8	0	1
J12.8	8	8	0	1
J92.9	8	8	0	2
K29.1	8	8	0	2
K40.90	8	8	0	2
K81.8	8	8	0	2
M51.2	8	8	0	1
R50.0	8	8	0	1
K21.0	7	12	0.000011	2
Z43.0	7	12	0.000011	2
J30.4	7	9	0.000044	1
C38.2	7	7	0	1
D64.8	7	7	0	5

D75.9	7	7	0	4
D86.2	7	7	0	2
E05.9	7	7	0	5
E87.8	7	7	0	4
F03	7	7	0	5
H91.8	7	7	0	5
I20.9	7	7	0	4
I25.2	7	7	0	1
I25.3	7	7	0	0
I30.9	7	7	0	0
J90	7	7	0	4
K80.80	7	7	0	4
M47.86	7	7	0	1
N39.81	7	7	0	1
Y84.1	7	7	0	4
J15.9	6	8	0.000013	1
C32.1	6	6	0	2
C32.8	6	6	0	2
G30.9	6	6	0	2
H26.9	6	6	0	2
I71.9	6	6	0	1
I84.9	6	6	0	1
J10.0	6	6	0	0
J30.3	6	6	0	1
J85.1	6	6	0	2
K58.9	6	6	0	2
R00.0	6	6	0	1
J01.9	5	5	0	1
J11.0	5	5	0	1
J45.1	5	5	0	1

Table S3 J46 edge list, ordered based on weight value, highest to lowest

Source	Target	Weight
J96.0	J46	40
I10	J46	31
J45.8	J46	27
I10	J96.0	20
J18.9	J46	20
J96.1	J46	18
J96.0	J45.8	17
N39.0	J46	16
J18.9	J45.8	14
J18.9	J96.0	14
I10	J45.8	13
N39.0	J96.0	12
B37.0	J46	11

J18.9	I10	10
J46	E66.0	10
J96.1	J45.8	10
I11.0	J46	9
J96.0	B37.0	9
I10	J96.1	8
I34.0	J46	8
I50.0	J46	8
J18.9	N39.0	8
J31.0	J46	8
N39.0	J45.8	8
I10	I11.0	7
I11.0	J45.8	7
I25.5	J46	7
J31.0	J96.0	7
J45.9	J46	7
J84.9	J46	7
J96.0	E66.0	7
J96.0	I11.0	7
J96.0	J45.9	7
E78.0	J46	6
I10	E66.0	6
I10	I25.5	6
I10	I34.0	6
I10	N39.0	6
I25.5	J45.8	6
J15.8	J46	6
J18.9	E66.0	6
J18.9	J96.1	6
J31.0	J18.9	6
J31.0	J45.8	6
J44.0	J46	6
B96.88	J45.8	5
B96.88	J46	5
I10	I50.0	5
I25.9	J46	5
J18.9	I11.0	5
J31.0	N39.0	5
J45.8	E66.0	5
J46	J44.1	5
J96.0	I34.0	5
J96.1	J44.1	5
R74.0	J46	5
Z72.0	J46	5
B37.1	I11.0	4
B37.1	J45.8	4

B37.1	J46	4
B90.9	J46	4
D53.9	J46	4
E78.0	J96.1	4
I10	B37.0	4
I10	J84.9	4
I10	K76.0	4
I10	Z99.1	4
I11.0	E66.0	4
I25.9	I10	4
I34.0	J45.8	4
J18.9	M47.84	4
J43.9	J46	4
J45.8	I50.0	4
J46	M47.84	4
J96.0	I50.0	4
J96.0	M47.84	4
J96.1	I25.5	4
K76.0	J46	4
R73	J46	4
R73	J96.0	4
R74.0	J96.0	4
Z11.5	J46	4
Z11.5	J96.0	4
Z99.1	J46	4
B37.1	J96.0	3
B96.88	I10	3
B96.88	I25.5	3
B96.88	J18.9	3
B96.88	J96.1	3
B96.88	N39.0	3
E11.65	J46	3
E78.0	J44.1	3
I10	B37.1	3
I10	E11.65	3
I10	I50.9	3
I10	J44.1	3
I10	J45.9	3
I10	M47.84	3
I11.0	I50.0	3
I25.5	I50.0	3
I25.9	J84.9	3
I50.0	E66.0	3
I50.9	J46	3
J18.9	B37.0	3
J18.9	B37.1	3

J18.9	I25.5	3
J31.0	I10	3
J44.0	J43.9	3
J96.0	D53.9	3
J96.0	I25.5	3
J96.0	J84.9	3
J96.0	Z72.0	3
J96.0	Z99.1	3
J96.1	B90.9	3
J96.1	I34.0	3
J96.1	I50.0	3
J96.1	J44.0	3
M54.4	J46	3
M54.4	J96.1	3
N18.90	I10	3
N18.90	J46	3
N39.0	B37.0	3
N39.0	I25.5	3
N39.0	J15.8	3
N39.0	J96.1	3
R73	I10	3
Z11.5	I10	3
Z72.0	J45.8	3
A41.8	J45.8	2
A41.8	J46	2
A41.8	J96.1	2
B37.0	I34.0	2
B37.0	J44.0	2
B37.0	Z72.0	2
B37.1	E66.8	2
B90.9	J47	2
B96.88	I11.9	2
B96.88	J31.0	2
B96.88	J96.0	2
D50.8	J45.8	2
D50.8	J46	2
D53.9	J84.9	2
E09.01	J44.0	2
E09.01	J46	2
E09.01	M54.4	2
E11.65	J45.8	2
E11.8	J15.8	2
E11.8	J46	2
E11.8	J96.0	2
E11.8	N39.0	2
E66.0	E11.9	2

E66.0	E79.0	2
E66.0	M47.84	2
E66.8	J45.8	2
E66.8	J46	2
E66.9	J46	2
E66.9	J84.9	2
E78.0	I34.0	2
E78.0	J45.8	2
E78.0	J96.0	2
E78.0	K21.0	2
E78.0	Z43.0	2
E87.6	J46	2
G20	J46	2
H91.9	J45.8	2
H91.9	J46	2
H91.9	J96.1	2
I10	D53.9	2
I10	E11.9	2
I10	E78.0	2
I10	E79.0	2
I10	E87.6	2
I10	G20	2
I10	I11.9	2
I10	I20.0	2
I10	I27.2	2
I10	J15.8	2
I10	J34.2	2
I10	J44.0	2
I10	J84.1	2
I10	K29.5	2
I10	Z71.3	2
I10	Z72.0	2
I11.0	E11.9	2
I11.0	E66.8	2
I11.0	I48	2
I11.0	J15.8	2
I11.0	J96.1	2
I11.0	M47.84	2
I11.0	Z71.3	2
I11.9	I25.5	2
I11.9	J45.8	2
I11.9	J46	2
I20.0	J46	2
I25.5	E66.0	2
I25.5	I83.1	2
I25.5	K81.1	2

I25.9	I50.0	2
I25.9	I50.9	2
I25.9	J96.0	2
I27.2	J46	2
I34.0	I25.5	2
I34.0	I27.2	2
I34.0	Z72.0	2
I50.9	J84.9	2
I50.9	J96.0	2
I87.2	J18.9	2
I87.2	J46	2
I87.2	J96.0	2
I87.2	N39.0	2
J15.8	J45.8	2
J15.9	J46	2
J15.9	J96.0	2
J18.0	E66.0	2
J18.9	A41.8	2
J18.9	I11.9	2
J18.9	I34.0	2
J18.9	I50.0	2
J18.9	L30.9	2
J18.9	R64	2
J18.9	Z71.3	2
J18.9	Z72.0	2
J30.4	J46	2
J30.4	J96.0	2
J31.0	E66.0	2
J31.0	K76.0	2
J31.0	L30.9	2
J31.0	R64	2
J34.2	I34.0	2
J34.2	J46	2
J34.2	J96.0	2
J43.9	J96.1	2
J44.9	J46	2
J45.0	J46	2
J45.8	B90.9	2
J45.8	I20.0	2
J45.9	E66.0	2
J46	E03.9	2
J46	E11.9	2
J46	E79.0	2
J46	E87.1	2
J46	I48	2
J46	I83.1	2

J46	J18.0	2
J47	J46	2
J84.1	J46	2
J84.9	E09.01	2
J84.9	J44.0	2
J84.9	M54.4	2
J96.0	E03.9	2
J96.0	E11.9	2
J96.0	E87.1	2
J96.0	E87.6	2
J96.0	J15.8	2
J96.0	J43.9	2
J96.0	J44.0	2
J96.0	J45.0	2
J96.0	K76.0	2
J96.0	L30.9	2
J96.0	M47.82	2
J96.0	T78.4	2
J96.0	Z71.3	2
J96.1	E09.01	2
J96.1	E66.0	2
J96.1	I11.9	2
J96.1	J15.8	2
J96.1	J47	2
J96.1	J84.9	2
J96.1	K81.1	2
J96.1	N40	2
J96.1	Z43.0	2
J96.1	Z72.0	2
K21.0	J44.1	2
K21.0	J46	2
K21.0	J96.1	2
K21.0	Z43.0	2
K25.9	E09.01	2
K25.9	J44.0	2
K25.9	J46	2
K25.9	J84.9	2
K25.9	J96.1	2
K25.9	M54.4	2
K25.9	N40	2
K29.5	J46	2
K76.0	I25.5	2
K81.1	J45.8	2
K81.1	J46	2
L30.9	J45.8	2
L30.9	J46	2

M47.82	J45.8	2
M47.82	J46	2
M47.84	J45.8	2
M54.4	J44.0	2
N18.90	I50.9	2
N39.0	E66.0	2
N39.0	I34.0	2
N39.0	I50.0	2
N39.0	J45.0	2
N39.0	J45.9	2
N39.0	K76.0	2
N39.0	M47.84	2
N39.0	Z72.0	2
N40	E09.01	2
N40	J44.0	2
N40	J46	2
N40	J84.9	2
N40	M54.4	2
R64	J45.8	2
R64	J46	2
R64	J96.0	2
R64	L30.9	2
R73	I25.5	2
R73	J45.8	2
R73	N39.0	2
R74.0	I10	2
R74.0	J18.9	2
T78.4	J46	2
Z11.5	J45.8	2
Z11.5	Z99.1	2
Z43.0	J44.1	2
Z43.0	J46	2
Z71.3	E66.0	2
Z71.3	J45.8	2
Z71.3	J46	2
Z99.1	E66.0	2
Z99.1	E79.0	2
A41.8	B95.6	1
A41.8	H54.0	1
A41.8	I34.0	1
A41.8	K40.90	1
B37.0	D53.9	1
B37.0	D64.9	1
B37.0	D75.9	1
B37.0	E66.0	1
B37.0	I50.0	1

B37.0	J15.8	1
B37.0	J45.0	1
B37.0	J45.1	1
B37.0	J45.8	1
B37.0	J82	1
B37.0	J84.9	1
B37.0	K51.9	1
B37.0	M47.84	1
B37.0	M51.2	1
B37.0	T78.4	1
B37.0	Z99.1	1
B37.1	B96.2	1
B37.1	E03.9	1
B37.1	E66.0	1
B37.1	H81.4	1
B37.1	H91.9	1
B37.1	I48	1
B37.1	I50.0	1
B37.1	J15.8	1
B37.1	J96.1	1
B37.1	M47.82	1
B37.1	M47.84	1
B37.1	Z71.3	1
B37.81	J44.0	1
B37.81	J46	1
B95.6	H54.0	1
B95.6	J45.8	1
B95.6	J46	1
B95.7	I83.1	1
B96.2	E66.8	1
B96.2	I50.0	1
B96.2	J45.8	1
B96.2	J46	1
B96.5	B90.9	1
B96.5	J46	1
B96.5	J47	1
B96.88	D50.8	1
B96.88	E11.65	1
B96.88	E11.8	1
B96.88	E66.0	1
B96.88	G20	1
B96.88	I07.1	1
B96.88	I11.0	1
B96.88	I25.6	1
B96.88	I34.0	1
B96.88	I50.0	1

B96.88	I70.0	1
B96.88	J15.8	1
B96.88	J91*	1
B96.88	K29.9	1
B96.88	K76.0	1
B96.88	K81.1	1
B96.88	K91.1	1
B96.88	K92.8	1
B96.88	L30.9	1
B96.88	M05.99	1
B96.88	M15.9	1
B96.88	M17.9	1
B96.88	M54.4	1
B96.88	R64	1
B96.88	Y43.4	1
B97.1	E66.0	1
B97.1	J45.8	1
B97.1	J46	1
C32.1	E78.0	1
C32.1	J44.1	1
C32.1	J46	1
C32.1	J96.1	1
C32.1	K21.0	1
C32.1	Z43.0	1
C32.8	J46	1
C34.9	E07.8	1
C34.9	I27.2	1
C34.9	J46	1
C34.9	K44.9	1
C34.9	Z72.0	1
C38.2	B37.0	1
C38.2	E66.0	1
C38.2	I50.0	1
C38.2	J44.0	1
C38.2	J46	1
C78.1	C34.9	1
C78.1	E07.8	1
C78.1	I27.2	1
C78.1	I34.0	1
C78.1	J46	1
C78.1	J84.1	1
C78.1	J96.1	1
C78.1	K44.9	1
C78.1	R59.0	1
C78.1	Z72.0	1
D50.8	A41.8	1

D50.8	E11.8	1
D50.8	E78.0	1
D50.8	I11.0	1
D50.8	I34.0	1
D50.8	J15.8	1
D50.8	J91*	1
D50.8	J96.0	1
D50.8	J96.1	1
D50.8	K81.8	1
D50.8	K91.1	1
D50.8	N39.0	1
D53.9	B90.9	1
D53.9	E11.65	1
D53.9	J90	1
D64.8	H91.8	1
D64.8	I50.0	1
D64.8	J46	1
D64.9	J46	1
D69.0	J45.0	1
D69.0	J46	1
D69.0	T78.2	1
D69.0	Z88.1	1
D69.0	Z88.6	1
D69.6	B90.9	1
D69.6	J18.9	1
D69.6	J45.8	1
D69.6	J46	1
D69.6	J96.1	1
D69.6	K29.1	1
D69.6	N39.0	1
D69.6	Z72.0	1
D72.8	E78.2	1
D72.8	I50.9	1
D72.8	J46	1
D72.8	J84.9	1
D72.8	K76.9	1
D72.8	Q61.3	1
D75.2	E11.9	1
D75.2	E66.0	1
D75.2	E79.0	1
D75.2	G20	1
D75.2	I25.0	1
D75.2	I44.7	1
D75.2	I48	1
D75.2	J46	1
D75.2	M47.84	1

D75.2	Z99.1	1
D75.9	J46	1
D86.2	D53.9	1
D86.2	I10	1
D86.2	I50.9	1
D86.2	J46	1
D86.2	J84.9	1
D86.2	J96.0	1
E03.9	E87.1	1
E03.9	I71.9	1
E04.9	E11.9	1
E04.9	E66.0	1
E04.9	I25.5	1
E04.9	I50.0	1
E04.9	I83.1	1
E04.9	J46	1
E05.9	D64.8	1
E05.9	F03	1
E05.9	H91.8	1
E05.9	I50.0	1
E05.9	J43.9	1
E05.9	J46	1
E05.9	J96.0	1
E06.3	B95.7	1
E06.3	F33.8	1
E06.3	I25.5	1
E06.3	I83.1	1
E06.3	I87.8	1
E06.3	J45.8	1
E06.3	J46	1
E06.3	J96.1	1
E06.3	K81.1	1
E06.3	M17.0	1
E07.8	I27.2	1
E07.8	J46	1
E07.8	K44.9	1
E09.01	B37.81	1
E11.65	G20	1
E11.65	I25.5	1
E11.65	J90	1
E11.65	K81.1	1
E11.8	I11.0	1
E11.8	J45.8	1
E11.8	J45.9	1
E11.8	J91*	1
E11.8	K91.1	1

E11.8	N39.81	1
E11.9	I50.0	1
E11.9	I83.1	1
E66.0	I25.0	1
E66.0	I44.7	1
E66.0	I48	1
E66.0	I83.1	1
E66.8	E03.9	1
E66.8	I50.0	1
E66.9	E09.01	1
E66.9	I10	1
E66.9	J44.0	1
E66.9	J96.1	1
E66.9	K25.9	1
E66.9	K76.0	1
E66.9	M54.4	1
E66.9	N40	1
E78.0	A41.8	1
E78.0	B90.9	1
E78.0	B96.5	1
E78.0	C32.8	1
E78.0	I25.5	1
E78.0	I50.0	1
E78.0	J45.9	1
E78.0	J47	1
E79.0	E11.9	1
E79.0	I44.7	1
E79.0	I48	1
E79.0	M47.84	1
E87.1	D75.2	1
E87.1	E11.9	1
E87.1	E66.0	1
E87.1	E79.0	1
E87.1	G20	1
E87.1	I11.0	1
E87.1	I25.0	1
E87.1	I44.7	1
E87.1	I48	1
E87.1	I50.9	1
E87.1	J18.9	1
E87.1	M47.84	1
E87.1	Z99.1	1
E87.6	D75.2	1
E87.6	E11.9	1
E87.6	E66.0	1
E87.6	E79.0	1

E87.6	E87.1	1
E87.6	G20	1
E87.6	I11.0	1
E87.6	I25.0	1
E87.6	I44.7	1
E87.6	I48	1
E87.6	I50.9	1
E87.6	J10.0	1
E87.6	J18.9	1
E87.6	M47.84	1
E87.6	Z99.1	1
E87.8	B37.0	1
E87.8	D75.9	1
E87.8	I20.9	1
E87.8	J15.8	1
E87.8	J46	1
E87.8	K80.80	1
E87.8	N39.0	1
F03	D64.8	1
F03	H91.8	1
F03	I50.0	1
F03	J43.9	1
F03	J46	1
F03	J96.0	1
F33.8	B95.7	1
F33.8	I83.1	1
F33.8	I87.8	1
F33.8	K81.1	1
G20	E11.9	1
G20	E66.0	1
G20	E79.0	1
G20	I25.0	1
G20	I44.7	1
G20	I48	1
G20	M47.84	1
G30.9	B90.9	1
G30.9	D53.9	1
G30.9	J46	1
H05.2	B37.0	1
H05.2	J44.0	1
H05.2	J45.8	1
H05.2	J46	1
H05.2	J96.0	1
H05.2	Q33.1	1
H05.2	Z72.0	1
H05.2	Z99.1	1

H10.9	H35.9	1
H10.9	I11.0	1
H10.9	I20.0	1
H10.9	I50.0	1
H10.9	J44.1	1
H10.9	J45.8	1
H10.9	J46	1
H10.9	J96.1	1
H26.9	J43.9	1
H26.9	J44.0	1
H26.9	J46	1
H26.9	J85.1	1
H26.9	J96.1	1
H35.9	I20.0	1
H35.9	I50.0	1
H35.9	J45.8	1
H35.9	J46	1
H81.4	I11.0	1
H81.4	J45.8	1
H81.4	J46	1
H81.4	M47.82	1
H81.4	M47.84	1
H91.8	I50.0	1
H91.8	J46	1
H91.9	B90.9	1
H91.9	B96.2	1
H91.9	E66.8	1
H91.9	I11.0	1
H91.9	I48	1
H91.9	I50.0	1
H91.9	J15.8	1
H91.9	J47	1
I07.1	E11.65	1
I07.1	G20	1
I07.1	I25.5	1
I07.1	I34.0	1
I07.1	J45.8	1
I07.1	J46	1
I07.1	K81.1	1
I08.0	B37.1	1
I08.0	B96.2	1
I08.0	E66.8	1
I08.0	H91.9	1
I08.0	I11.0	1
I08.0	I48	1
I08.0	I50.0	1

I08.0	J15.8	1
I08.0	J45.8	1
I08.0	J46	1
I08.0	J96.1	1
I10	B90.9	1
I10	B96.5	1
I10	B97.1	1
I10	C34.9	1
I10	C78.1	1
I10	D72.8	1
I10	D75.2	1
I10	E03.9	1
I10	E04.9	1
I10	E07.8	1
I10	E11.8	1
I10	E66.8	1
I10	E78.2	1
I10	E87.1	1
I10	H05.2	1
I10	H10.9	1
I10	H26.9	1
I10	H35.9	1
I10	H81.4	1
I10	I07.1	1
I10	I25.0	1
I10	I25.3	1
I10	I25.6	1
I10	I30.9	1
I10	I36.0	1
I10	I44.7	1
I10	I48	1
I10	I51.7	1
I10	I70.0	1
I10	I83.1	1
I10	J10.0	1
I10	J12.9	1
I10	J18.0	1
I10	J30.3	1
I10	J32.1	1
I10	J43.9	1
I10	J44.9	1
I10	J45.0	1
I10	J47	1
I10	J82	1
I10	J85.1	1
I10	J90	1

I10	J95.1	1
I10	K29.9	1
I10	K44.9	1
I10	K51.9	1
I10	K76.9	1
I10	K81.1	1
I10	K92.8	1
I10	M15.9	1
I10	M17.9	1
I10	M30.1	1
I10	M47.82	1
I10	M54.4	1
I10	M79.28	1
I10	N11.0	1
I10	N39.81	1
I10	Q33.1	1
I10	Q61.3	1
I10	R59.0	1
I10	Y43.4	1
I10	Y84.1	1
I11.0	B96.2	1
I11.0	B97.1	1
I11.0	D75.2	1
I11.0	E03.9	1
I11.0	E04.9	1
I11.0	E79.0	1
I11.0	G20	1
I11.0	H35.9	1
I11.0	I20.0	1
I11.0	I25.0	1
I11.0	I25.5	1
I11.0	I44.7	1
I11.0	I83.1	1
I11.0	J44.1	1
I11.0	J91*	1
I11.0	K91.1	1
I11.0	M47.82	1
I11.0	Z99.1	1
I11.9	E66.0	1
I11.9	I50.0	1
I11.9	K29.9	1
I11.9	K92.8	1
I11.9	M15.9	1
I11.9	Y43.4	1
I20.0	I50.0	1
I20.8	E78.0	1

I20.8	I10	1
I20.8	I25.5	1
I20.8	I34.0	1
I20.8	I50.0	1
I20.8	J45.8	1
I20.8	J46	1
I20.8	J96.0	1
I20.8	N39.0	1
I20.8	R73	1
I20.9	B37.0	1
I20.9	D75.9	1
I20.9	J15.8	1
I20.9	J46	1
I25.0	E11.9	1
I25.0	E79.0	1
I25.0	I44.7	1
I25.0	I48	1
I25.0	M47.84	1
I25.2	B37.0	1
I25.2	C38.2	1
I25.2	E66.0	1
I25.2	I50.0	1
I25.2	J44.0	1
I25.2	J46	1
I25.3	J46	1
I25.3	J84.9	1
I25.3	Z99.1	1
I25.5	B95.7	1
I25.5	E11.9	1
I25.5	F33.8	1
I25.5	G20	1
I25.5	I20.0	1
I25.5	I87.8	1
I25.5	K29.9	1
I25.5	K92.8	1
I25.5	N11.0	1
I25.6	E11.65	1
I25.6	G20	1
I25.6	I07.1	1
I25.6	I25.5	1
I25.6	I34.0	1
I25.6	J45.8	1
I25.6	J46	1
I25.6	K81.1	1
I25.9	B37.0	1
I25.9	C38.2	1

I25.9	D53.9	1
I25.9	D72.8	1
I25.9	D86.2	1
I25.9	E66.0	1
I25.9	E66.9	1
I25.9	E78.2	1
I25.9	I25.2	1
I25.9	J18.9	1
I25.9	J44.0	1
I25.9	K76.0	1
I25.9	K76.9	1
I25.9	N18.90	1
I25.9	N39.0	1
I25.9	Q61.3	1
I25.9	R74.0	1
I27.2	J45.8	1
I30.9	I25.3	1
I30.9	J46	1
I30.9	J84.9	1
I30.9	J96.0	1
I30.9	Z99.1	1
I34.0	C34.9	1
I34.0	D64.9	1
I34.0	E07.8	1
I34.0	E11.65	1
I34.0	G20	1
I34.0	I50.0	1
I34.0	J45.0	1
I34.0	J82	1
I34.0	K44.9	1
I34.0	K51.9	1
I34.0	K81.1	1
I36.0	B37.0	1
I36.0	I34.0	1
I36.0	J45.0	1
I36.0	J46	1
I36.0	J82	1
I36.0	K51.9	1
I44.7	E11.9	1
I44.7	M47.84	1
I48	B96.2	1
I48	E11.9	1
I48	E66.8	1
I48	I44.7	1
I48	I50.0	1
I48	J15.8	1

I48	J45.8	1
I48	J96.1	1
I48	M47.84	1
I49.3	J46	1
I50.0	I83.1	1
I50.0	J44.0	1
I50.9	D53.9	1
I50.9	D75.2	1
I50.9	E11.9	1
I50.9	E66.0	1
I50.9	E78.2	1
I50.9	E79.0	1
I50.9	G20	1
I50.9	I11.0	1
I50.9	I25.0	1
I50.9	I44.7	1
I50.9	I48	1
I50.9	K76.9	1
I50.9	M47.84	1
I50.9	Q61.3	1
I50.9	Z99.1	1
I51.7	D75.2	1
I51.7	E11.9	1
I51.7	E66.0	1
I51.7	E79.0	1
I51.7	E87.1	1
I51.7	E87.6	1
I51.7	G20	1
I51.7	I11.0	1
I51.7	I25.0	1
I51.7	I44.7	1
I51.7	I48	1
I51.7	I50.9	1
I51.7	J18.9	1
I51.7	J46	1
I51.7	J96.0	1
I51.7	M47.84	1
I51.7	Z99.1	1
I70.0	E66.0	1
I70.0	I11.9	1
I70.0	I25.5	1
I70.0	I50.0	1
I70.0	J45.8	1
I70.0	J46	1
I70.0	J96.1	1
I70.0	K29.9	1

I70.0	K92.8	1
I70.0	M15.9	1
I71.9	E87.1	1
I84.9	J45.8	1
I84.9	J46	1
I87.2	B37.0	1
I87.2	E66.0	1
I87.2	I10	1
I87.2	J31.0	1
I87.2	J45.9	1
I87.2	M47.84	1
I87.8	B95.7	1
I87.8	I83.1	1
J01.9	J45.8	1
J01.9	J46	1
J01.9	M47.82	1
J10.0	J46	1
J11.0	E66.0	1
J11.0	J18.0	1
J11.0	J45.8	1
J11.0	J46	1
J12.8	I49.3	1
J12.8	J45.8	1
J12.8	J46	1
J12.8	T78.4	1
J12.8	Z72.0	1
J12.9	I20.0	1
J12.9	I25.5	1
J12.9	J45.8	1
J12.9	J46	1
J12.9	J84.1	1
J12.9	J96.0	1
J12.9	N11.0	1
J15.8	B37.81	1
J15.8	B96.2	1
J15.8	D53.9	1
J15.8	D75.9	1
J15.8	E09.01	1
J15.8	E11.65	1
J15.8	E66.8	1
J15.8	I50.0	1
J15.8	J44.0	1
J15.8	J84.9	1
J15.8	J90	1
J15.8	J91*	1
J15.8	K91.1	1

J15.8	M54.4	1
J15.8	N39.81	1
J15.8	N40	1
J15.9	B37.0	1
J18.0	E79.0	1
J18.0	J45.8	1
J18.9	B90.9	1
J18.9	B95.6	1
J18.9	B95.7	1
J18.9	B97.1	1
J18.9	D50.8	1
J18.9	D64.9	1
J18.9	D75.2	1
J18.9	E03.9	1
J18.9	E06.3	1
J18.9	E11.9	1
J18.9	E66.8	1
J18.9	E78.0	1
J18.9	E79.0	1
J18.9	F33.8	1
J18.9	G20	1
J18.9	H54.0	1
J18.9	H81.4	1
J18.9	I25.0	1
J18.9	I44.7	1
J18.9	I48	1
J18.9	I50.9	1
J18.9	I70.0	1
J18.9	I83.1	1
J18.9	I84.9	1
J18.9	I87.8	1
J18.9	J45.9	1
J18.9	J92.9	1
J18.9	K29.1	1
J18.9	K29.5	1
J18.9	K29.9	1
J18.9	K40.90	1
J18.9	K76.0	1
J18.9	K81.1	1
J18.9	K81.8	1
J18.9	K92.8	1
J18.9	M05.99	1
J18.9	M15.9	1
J18.9	M17.0	1
J18.9	M17.9	1
J18.9	M47.82	1

J18.9	M51.2	1
J18.9	R50.0	1
J18.9	Y43.4	1
J18.9	Z99.1	1
J30.3	I34.0	1
J30.3	J45.9	1
J30.3	J46	1
J30.3	J96.0	1
J30.4	B37.0	1
J30.4	J15.9	1
J30.4	J45.1	1
J30.4	N39.0	1
J30.4	T78.4	1
J31.0	B37.0	1
J31.0	B97.1	1
J31.0	I11.0	1
J31.0	I11.9	1
J31.0	I25.5	1
J31.0	I34.0	1
J31.0	I36.0	1
J31.0	I49.3	1
J31.0	I84.9	1
J31.0	J12.8	1
J31.0	J34.2	1
J31.0	J45.0	1
J31.0	J45.9	1
J31.0	J82	1
J31.0	J96.1	1
J31.0	K51.9	1
J31.0	M05.99	1
J31.0	M17.9	1
J31.0	M30.1	1
J31.0	T78.4	1
J31.0	Y43.4	1
J31.0	Z71.3	1
J31.0	Z72.0	1
J32.1	E04.9	1
J32.1	E11.9	1
J32.1	E66.0	1
J32.1	I11.0	1
J32.1	I25.5	1
J32.1	I50.0	1
J32.1	I83.1	1
J32.1	J45.9	1
J32.1	J46	1
J32.1	J96.0	1

J32.1	K76.0	1
J32.1	M79.28	1
J34.2	B37.0	1
J34.2	I36.0	1
J34.2	J30.3	1
J34.2	J45.0	1
J34.2	J45.9	1
J34.2	J82	1
J34.2	K51.9	1
J34.2	K76.0	1
J34.2	M30.1	1
J34.2	N39.0	1
J43.9	B37.81	1
J43.9	B90.9	1
J43.9	D53.9	1
J43.9	D64.8	1
J43.9	E09.01	1
J43.9	G30.9	1
J43.9	H91.8	1
J43.9	I50.0	1
J43.9	J15.8	1
J43.9	J84.9	1
J43.9	J85.1	1
J43.9	M54.4	1
J43.9	N40	1
J44.0	B90.9	1
J44.0	D53.9	1
J44.0	E66.0	1
J44.0	G30.9	1
J44.1	B90.9	1
J44.1	B96.5	1
J44.1	C32.8	1
J44.1	E66.0	1
J44.1	E79.0	1
J44.1	H35.9	1
J44.1	I20.0	1
J44.1	I50.0	1
J44.1	J18.0	1
J44.1	J45.8	1
J44.1	J47	1
J44.9	C34.9	1
J44.9	C78.1	1
J44.9	E03.9	1
J44.9	E07.8	1
J44.9	E87.1	1
J44.9	I27.2	1

J44.9	I34.0	1
J44.9	I71.9	1
J44.9	J45.9	1
J44.9	J84.1	1
J44.9	J96.0	1
J44.9	J96.1	1
J44.9	K29.5	1
J44.9	K44.9	1
J44.9	R59.0	1
J44.9	Z72.0	1
J45.0	J82	1
J45.0	K51.9	1
J45.1	J46	1
J45.1	T78.4	1
J45.8	B95.7	1
J45.8	E03.9	1
J45.8	F33.8	1
J45.8	G20	1
J45.8	H54.0	1
J45.8	I49.3	1
J45.8	I83.1	1
J45.8	I87.8	1
J45.8	J44.0	1
J45.8	J47	1
J45.9	B37.0	1
J45.9	E03.9	1
J45.9	E04.9	1
J45.9	E11.9	1
J45.9	E87.1	1
J45.9	I11.0	1
J45.9	I25.5	1
J45.9	I34.0	1
J45.9	I50.0	1
J45.9	I71.9	1
J45.9	I83.1	1
J45.9	J15.8	1
J45.9	N39.81	1
J46	B95.7	1
J46	E78.2	1
J46	F33.8	1
J46	H54.0	1
J46	I25.0	1
J46	I44.7	1
J46	I71.9	1
J46	I87.8	1
J46	K76.9	1

J82	J46	1
J84.1	C34.9	1
J84.1	E07.8	1
J84.1	I20.0	1
J84.1	I25.5	1
J84.1	I27.2	1
J84.1	I34.0	1
J84.1	J45.8	1
J84.1	J96.1	1
J84.1	K44.9	1
J84.1	N11.0	1
J84.1	R59.0	1
J84.1	Z72.0	1
J84.9	B37.81	1
J84.9	E78.2	1
J84.9	K76.9	1
J85.1	J44.0	1
J85.1	J46	1
J90	J46	1
J91*	J45.8	1
J91*	J46	1
J91*	K91.1	1
J92.9	A41.8	1
J92.9	B95.6	1
J92.9	H54.0	1
J92.9	J45.8	1
J92.9	J46	1
J92.9	J96.1	1
J92.9	K40.90	1
J94.1	B90.9	1
J94.1	B96.5	1
J94.1	E78.0	1
J94.1	I10	1
J94.1	J44.1	1
J94.1	J46	1
J94.1	J47	1
J94.1	J95.1	1
J94.1	J96.1	1
J95.1	B90.9	1
J95.1	B96.5	1
J95.1	E78.0	1
J95.1	J44.1	1
J95.1	J46	1
J95.1	J47	1
J95.1	J96.1	1
J96.0	B90.9	1

J96.0	B97.1	1
J96.0	D64.8	1
J96.0	D64.9	1
J96.0	D69.0	1
J96.0	D75.2	1
J96.0	E04.9	1
J96.0	E11.65	1
J96.0	E66.8	1
J96.0	E79.0	1
J96.0	G20	1
J96.0	G30.9	1
J96.0	H81.4	1
J96.0	H91.8	1
J96.0	I20.0	1
J96.0	I25.0	1
J96.0	I25.3	1
J96.0	I27.2	1
J96.0	I36.0	1
J96.0	I44.7	1
J96.0	I48	1
J96.0	I49.3	1
J96.0	I71.9	1
J96.0	I83.1	1
J96.0	I84.9	1
J96.0	J01.9	1
J96.0	J10.0	1
J96.0	J11.0	1
J96.0	J12.8	1
J96.0	J18.0	1
J96.0	J45.1	1
J96.0	J82	1
J96.0	J84.1	1
J96.0	J91*	1
J96.0	K51.9	1
J96.0	K91.1	1
J96.0	M30.1	1
J96.0	M51.2	1
J96.0	N11.0	1
J96.0	N39.81	1
J96.0	Q33.1	1
J96.0	T78.2	1
J96.0	Z88.1	1
J96.0	Z88.6	1
J96.1	B37.81	1
J96.1	B95.6	1
J96.1	B95.7	1

J96.1	B96.2	1
J96.1	B96.5	1
J96.1	C32.8	1
J96.1	C34.9	1
J96.1	E07.8	1
J96.1	E11.65	1
J96.1	E66.8	1
J96.1	E79.0	1
J96.1	F33.8	1
J96.1	G20	1
J96.1	H35.9	1
J96.1	H54.0	1
J96.1	I07.1	1
J96.1	I20.0	1
J96.1	I25.6	1
J96.1	I27.2	1
J96.1	I83.1	1
J96.1	I87.8	1
J96.1	J18.0	1
J96.1	J85.1	1
J96.1	K29.1	1
J96.1	K29.9	1
J96.1	K40.90	1
J96.1	K44.9	1
J96.1	K92.8	1
J96.1	M15.9	1
J96.1	Y43.4	1
J96.1	Z99.1	1
K21.0	C32.8	1
K25.9	B37.81	1
K25.9	J15.8	1
K25.9	J43.9	1
K29.1	B90.9	1
K29.1	J45.8	1
K29.1	J46	1
K29.5	B37.0	1
K29.5	C34.9	1
K29.5	C78.1	1
K29.5	E07.8	1
K29.5	I27.2	1
K29.5	I34.0	1
K29.5	J84.1	1
K29.5	J96.0	1
K29.5	J96.1	1
K29.5	K44.9	1
K29.5	R59.0	1

K29.5	Z72.0	1
K29.9	E66.0	1
K29.9	I50.0	1
K29.9	J45.8	1
K29.9	J46	1
K40.90	B95.6	1
K40.90	H54.0	1
K40.90	J45.8	1
K40.90	J46	1
K44.9	I27.2	1
K44.9	J46	1
K51.9	J46	1
K51.9	J82	1
K58.9	B90.9	1
K58.9	H91.9	1
K58.9	J45.8	1
K58.9	J46	1
K58.9	J47	1
K58.9	J96.1	1
K76.0	B37.0	1
K76.0	E04.9	1
K76.0	E11.9	1
K76.0	E66.0	1
K76.0	I11.0	1
K76.0	I11.9	1
K76.0	I34.0	1
K76.0	I36.0	1
K76.0	I50.0	1
K76.0	I83.1	1
K76.0	J45.0	1
K76.0	J45.8	1
K76.0	J45.9	1
K76.0	J82	1
K76.0	J84.9	1
K76.0	J96.1	1
K76.0	K51.9	1
K76.0	M30.1	1
K76.0	Y43.4	1
K76.9	E78.2	1
K80.80	B37.0	1
K80.80	D75.9	1
K80.80	I20.9	1
K80.80	J15.8	1
K80.80	J46	1
K80.80	N39.0	1
K81.1	B95.7	1

K81.1	G20	1
K81.1	I83.1	1
K81.1	I87.8	1
K81.8	A41.8	1
K81.8	E78.0	1
K81.8	I34.0	1
K81.8	J45.8	1
K81.8	J46	1
K81.8	J96.1	1
K91.1	J45.8	1
K91.1	J46	1
K92.8	E66.0	1
K92.8	I50.0	1
K92.8	J45.8	1
K92.8	J46	1
K92.8	K29.9	1
M05.99	I10	1
M05.99	I11.9	1
M05.99	I25.5	1
M05.99	J45.8	1
M05.99	J46	1
M05.99	J96.1	1
M05.99	K76.0	1
M05.99	M17.9	1
M05.99	N39.0	1
M05.99	Y43.4	1
M15.9	E66.0	1
M15.9	I25.5	1
M15.9	I50.0	1
M15.9	J45.8	1
M15.9	J46	1
M15.9	K29.9	1
M15.9	K92.8	1
M17.0	B95.7	1
M17.0	F33.8	1
M17.0	I25.5	1
M17.0	I83.1	1
M17.0	I87.8	1
M17.0	J45.8	1
M17.0	J46	1
M17.0	J96.1	1
M17.0	K81.1	1
M17.9	I11.9	1
M17.9	I25.5	1
M17.9	J45.8	1
M17.9	J46	1

M17.9	J96.1	1
M17.9	K76.0	1
M17.9	Y43.4	1
M30.1	B37.0	1
M30.1	I34.0	1
M30.1	I36.0	1
M30.1	J45.0	1
M30.1	J46	1
M30.1	J82	1
M30.1	K51.9	1
M47.84	E11.9	1
M47.84	M47.82	1
M47.86	E66.0	1
M47.86	J18.9	1
M47.86	J45.8	1
M47.86	J46	1
M47.86	J96.0	1
M47.86	M47.84	1
M47.86	N39.0	1
M51.2	D64.9	1
M51.2	I34.0	1
M51.2	J46	1
M51.2	Z72.0	1
M54.4	B37.81	1
M54.4	E11.65	1
M54.4	G20	1
M54.4	I07.1	1
M54.4	I25.5	1
M54.4	I25.6	1
M54.4	I34.0	1
M54.4	J45.8	1
M54.4	K81.1	1
M79.28	E04.9	1
M79.28	E11.9	1
M79.28	E66.0	1
M79.28	I11.0	1
M79.28	I25.5	1
M79.28	I50.0	1
M79.28	I83.1	1
M79.28	J45.9	1
M79.28	J46	1
M79.28	J96.0	1
M79.28	K76.0	1
N11.0	I20.0	1
N11.0	J45.8	1
N11.0	J46	1

N18.90	D53.9	1
N18.90	D72.8	1
N18.90	D75.2	1
N18.90	E11.65	1
N18.90	E11.9	1
N18.90	E66.0	1
N18.90	E78.2	1
N18.90	E79.0	1
N18.90	E87.1	1
N18.90	E87.6	1
N18.90	G20	1
N18.90	I11.0	1
N18.90	I25.0	1
N18.90	I44.7	1
N18.90	I48	1
N18.90	I51.7	1
N18.90	J15.8	1
N18.90	J18.9	1
N18.90	J84.9	1
N18.90	J90	1
N18.90	J96.0	1
N18.90	K76.9	1
N18.90	M47.84	1
N18.90	Q61.3	1
N18.90	Y84.1	1
N18.90	Z99.1	1
N39.0	B90.9	1
N39.0	B95.7	1
N39.0	D69.0	1
N39.0	D75.9	1
N39.0	E06.3	1
N39.0	E78.0	1
N39.0	F33.8	1
N39.0	I11.0	1
N39.0	I11.9	1
N39.0	I20.9	1
N39.0	I36.0	1
N39.0	I49.3	1
N39.0	I83.1	1
N39.0	I87.8	1
N39.0	J12.8	1
N39.0	J15.9	1
N39.0	J82	1
N39.0	J91*	1
N39.0	K29.1	1
N39.0	K51.9	1

N39.0	K81.1	1
N39.0	K91.1	1
N39.0	L30.9	1
N39.0	M17.0	1
N39.0	M17.9	1
N39.0	M30.1	1
N39.0	N39.81	1
N39.0	T78.2	1
N39.0	T78.4	1
N39.0	Y43.4	1
N39.0	Z88.1	1
N39.0	Z88.6	1
N39.81	J46	1
N40	B37.81	1
Q33.1	B37.0	1
Q33.1	J44.0	1
Q33.1	J45.8	1
Q33.1	J46	1
Q33.1	Z72.0	1
Q33.1	Z99.1	1
Q61.3	E78.2	1
Q61.3	J46	1
Q61.3	J84.9	1
Q61.3	K76.9	1
R00.0	I10	1
R00.0	I27.2	1
R00.0	I34.0	1
R00.0	J45.8	1
R00.0	J46	1
R00.0	J96.0	1
R31	I10	1
R31	I20.0	1
R31	I25.5	1
R31	J12.9	1
R31	J45.8	1
R31	J46	1
R31	J84.1	1
R31	J96.0	1
R31	N11.0	1
R31	R73	1
R50.0	B37.0	1
R50.0	D64.9	1
R50.0	I34.0	1
R50.0	J46	1
R50.0	J96.0	1
R50.0	M51.2	1

R50.0	Z72.0	1
R59.0	C34.9	1
R59.0	E07.8	1
R59.0	I27.2	1
R59.0	I34.0	1
R59.0	J46	1
R59.0	J96.1	1
R59.0	K44.9	1
R59.0	Z72.0	1
R64	N39.0	1
R73	D69.0	1
R73	E78.0	1
R73	E87.6	1
R73	I20.0	1
R73	I34.0	1
R73	I50.0	1
R73	J10.0	1
R73	J12.9	1
R73	J45.0	1
R73	J84.1	1
R73	N11.0	1
R73	T78.2	1
R73	Z88.1	1
R73	Z88.6	1
R74.0	B37.0	1
R74.0	D69.0	1
R74.0	D75.2	1
R74.0	E11.9	1
R74.0	E66.0	1
R74.0	E66.9	1
R74.0	E79.0	1
R74.0	E87.1	1
R74.0	E87.6	1
R74.0	G20	1
R74.0	I11.0	1
R74.0	I25.0	1
R74.0	I44.7	1
R74.0	I48	1
R74.0	I50.9	1
R74.0	I51.7	1
R74.0	I84.9	1
R74.0	J15.9	1
R74.0	J31.0	1
R74.0	J45.0	1
R74.0	J45.8	1
R74.0	J84.9	1

R74.0	K76.0	1
R74.0	M47.84	1
R74.0	N18.90	1
R74.0	N39.0	1
R74.0	R73	1
R74.0	T78.2	1
R74.0	Z88.1	1
R74.0	Z88.6	1
R74.0	Z99.1	1
T78.2	J45.0	1
T78.2	J46	1
T78.4	I49.3	1
T78.4	J45.8	1
Y43.4	I25.5	1
Y43.4	J45.8	1
Y43.4	J46	1
Y84.1	D53.9	1
Y84.1	E11.65	1
Y84.1	J15.8	1
Y84.1	J46	1
Y84.1	J90	1
Z11.5	B37.0	1
Z11.5	E87.6	1
Z11.5	H05.2	1
Z11.5	I25.3	1
Z11.5	I30.9	1
Z11.5	J01.9	1
Z11.5	J10.0	1
Z11.5	J44.0	1
Z11.5	J84.9	1
Z11.5	M47.82	1
Z11.5	Q33.1	1
Z11.5	R73	1
Z11.5	Z72.0	1
Z43.0	C32.8	1
Z71.3	B97.1	1
Z72.0	B90.9	1
Z72.0	D64.9	1
Z72.0	E07.8	1
Z72.0	I27.2	1
Z72.0	I49.3	1
Z72.0	J44.0	1
Z72.0	K29.1	1
Z72.0	K44.9	1
Z72.0	T78.4	1
Z72.0	Z99.1	1

Z88.1	J45.0	1
Z88.1	J46	1
Z88.1	T78.2	1
Z88.1	Z88.6	1
Z88.6	J45.0	1
Z88.6	J46	1
Z88.6	T78.2	1
Z99.1	E11.9	1
Z99.1	G20	1
Z99.1	I25.0	1
Z99.1	I44.7	1
Z99.1	I48	1
Z99.1	J18.0	1
Z99.1	J44.0	1
Z99.1	J44.1	1
Z99.1	J45.8	1
Z99.1	J84.9	1
Z99.1	M47.84	1
